# Supplementary material for: The use of social media as a two-way mirror for narcissistic adolescents from Austria, Belgium, South-Korea, and Spain
Source: PLoS One. 2022 Aug 31;17(8):e0272868. doi: 10.1371/journal.pone.0272868 (PMC9432699; doi:10.1371/journal.pone.0272868)
Supplement: Attachment — Submitted filename: Response to Reviewers.docx [file pone.0272868.s002.docx]

**REVIEWS**

We thank the reviewers for their relevant and useful comments. Reviewer #2 is addressed individually, with the reviewer’s comments in black font and our answers in blue font.

**Reviewer 2**

The authors should further explain the tables (e.g., what the columns represent, explain abbreviations, what does High and Low mean, what is threshold,

As explained next in each comment, some tables had wrong information or were not clear. Following the changes proposed by the reviewers, we have edited the tables, explained them in the text and have deleted two of the tables (7 and 10) so that the Results sections is much clearer now. We have provided the figures as editable PDFs files so that the quality is high.

The results are not properly explained. Some variables are not explained e.g. RQ, M. How they are obtained, what they represent. equations need to be provided

RQ is the Research Question (we deleted this mention on the Results because it could be misleading). M’s are means or averages calculated for each variable. We have reviewed the text in order to further specify to what variables refer each M value. In some cases, we have added the specific equation (correlation, student-t or ANOVA) that was used, just before the result in APA format, for clarification. As mentioned, Results have now a clearer structure and the reference to the tables information is more explicit so that they are much easier to follow.

Some values are presented in the text but are not provided in the table

NPI-13 was mentioned in the text but it was not in table 3. It has been added. We have also found that the mode was not informed in table 1, so it has also been added too. We have reviewed all the connections between tables and text.

With respect to the Result: Further interpretation is needed;

SD is not clear; Line 378, what are r, p, and how they are obtained?;

We have clarified these data and added that the test is a correlation test. We have made clear that the r is the correlation coefficient, this is, the strength and direction of the association of variables, and the p is how significant/relevant is this direction, accepted if under .05.

Line 388, what is RQ?; Line 389, what is M?; Line 399, “(t (1716) = 2.41, p = .016).” are these numbers presented in a table??

We deleted the RQ in this line since it is a mistake. M is “Mean” (or average) as per APA abbreviation. With regard to t test (previously in line 399), results are presented graphically in Figure 2. We have also noticed a mistake in the captions of the columns of table 2. We have amended it and we have further explained the value of Table 2. We believe that results of this part are now much easier to follow. Thanks.

Table 1: Not properly explained; It was not mentioned in the text; What does first column represent? What is FB years?;Where are the values for Mode Column?; What is N column?

We have tried to clarify and further explain Table 1. First, we have informed the category of first column, added the mode, and a footnote with the names of abbreviations at full. We have also made sure that Table 1 is mentioned in the Results section (line 378) and have placed after first paragraph of results so that it is fully connected with the text.

In table 2, why value of country is High and Low? What does it mean? On what basis they are considered Low and High??What is the threshold;

2nd column should be Gender (Mistake);

As mentioned earlier, the captions of the first and second columns in table 2 were wrong. “Country” has been substituted by “Narcissism”, “SNS use” by “Gender”, and “NPQCS” by “SNS use”. Regarding the high vs low threshold, we have added the information of the mean average and reported is as the threshold in table 2 as a footnote, in figure 2 and within the explanations in the text.

Line 404, All countries except South Korea reported positive correlations for H1 (all p-values <.005 except South Korea’s NPQC-R) ? is this shown in the table?

Yes, indeed; we have now included the significance of data for all tables with asterisks (*) and, int eh particular case of table 3, have amended the text to which these comments refer (changing “positive” for “significant”). Also, since South-Korea does not show positive correlations between social media use and the narcissism (NPQC-R), the ANOVA model that stems from these data does not consider data from this country (table 4).

Line 406, “Spanish teenagers reported the highest values of SNS use (Table 3), highest narcissism levels.” It is not the highest narcissism levels because South Korea is higher.

Yes, indeed. However, as shown in the previous comment, South Korea’s data were not statistically significant; having said this, we have rephrased the original sentence in order to make clear that we refer to statistically significant evidence.

Table 3,

in its caption, they mentioned NPI-13 but is it not in the table

How p-values are obtained?

NPI-13 data has been added in Table 3. And as mentioned, a footnote has been added to the table so that the type of test (correlation) and significance are now reported (*).

Line 415.” all p-values under .05, except South Korea)” please explain.

We have further clarified that South Korean adolescents did not show a significant correlation but we also highlight the fact that the other countries did show a significant correlations with narcissism.

Line 445, “Table 6. Social media use (SNS use) and self-appearance focused behavior use of SNS”, in other table captions SNS is mentioned without the complete phrase. Consistency is required.

We have kept the full name of variables in the titles of figures and tables (with the abbreviation in parenthesis so that the reader can easily see the equivalence when the abbreviation is used as a caption of a column or an axis of these figures and tables). Within the text, abbreviations are only used when the word is repeated in the same paragraph making sure the reader can connect it with the full word it is referring to.

Please explain Table 4

We have further developed the explanations that arise from Table 4 and Figure 3 with a more explicit illustration of the numbers in the table and the graphics in Figure 3. But, as explained, these explanations are limited and only reinforce previous data: the ANOVA model of Table 4 does not include South Korean because it stems from the high versus low narcissism divide as calculated with the mean of the NPQC-R index -being the case that South Korea had not shown correlation between social media use and NPQC-R.

Figure 1 is not mentioned in the text.

Figure 1 is highly relevant and clarifying figure in the article, so we appreciate the reviewer noticing this. The reference has been added to follow the connection of variables in the form of hypothesis and research question.

Fig 2, what is x-axis, what is y-axis?

We added the concepts being measured in the figure.

Figure 4, give full form of SCA should be added.

We added the full range of SCA and SNS use axes in figure 4.

For Figure 2,3, and 5, the threshold should be shown in the Figures

The thresholds for the variables in the three figures have been added to the very captions of those.
