# Supplementary material for: The use of social media as a two-way mirror for narcissistic adolescents from Austria, Belgium, South-Korea, and Spain
Source: PLoS One. 2022 Aug 31;17(8):e0272868. doi: 10.1371/journal.pone.0272868 (PMC9432699; doi:10.1371/journal.pone.0272868)
Supplement: Attachment — Submitted filename: Response to Reviewers1.docx [file pone.0272868.s003.docx]

**REVIEWS**

We thank the reviewer 2 for their relevant and useful comments. Reviewer #2 is addressed individually, with the reviewer’s comments in black font and our answers in blue font.

**Reviewer 2**

Table 1, still needs more explanation. What is N column? It is still not clear.

We see that N data is confusing in this table, so we remove it. The sample size is reported in the Method.

Line 376, “The overall mean (M) for NPQC-R was 3.6”. According to the contents in Table 1 it is 3.05??

We have double checked and the correct figure is 3.05. It has been amended. Thanks.

In the footnote for table 2, “The threshold to divide segments between high and low narcissism is the mean average of this variable, that is higher or lower than 3.05 (as also reported in Figure 2).”. Please double check.

We have double checked the average of NPQC-R and it is 3,05, not 3,6.

Table 7, in its caption, gender is mentioned but is it not in the table and not explained in the text.

We have double checked. This is a mistake in the caption; “Gender” has been removed from the caption. Thanks a lot.
